# Molecular markers of dihydroartemisinin-piperaquine resistance in northwestern Thailand

**DOI:** 10.1186/s12936-022-04382-5

**Published:** 2022-11-27

**Authors:** Khine Nwe Win, Khajohnpong Manopwisedjaroen, Kanit Phumchuea, Chayanut Suansomjit, Kesinee Chotivanich, Saranath Lawpoolsri, Liwang Cui, Jetsumon Sattabongkot, Wang Nguitragool

**Affiliations:** 1 Department of Tropical Hygiene, Faculty of Tropical Medicine, Mahidol University, Bangkok, 10400 Thailand; 2 Mahidol Vivax Research Unit, Faculty of Tropical Medicine, Mahidol University, Bangkok, 10400 Thailand; 3 Department of Clinical Tropical Medicine, Faculty of Tropical Medicine, Mahidol University, Bangkok, 10400 Thailand; 4 Department of Internal Medicine, Morsani College of Medicine, University of South Florida, Tampa, FL 33612 USA; 5 Department of Molecular Tropical Medicine and Genetics, Faculty of Tropical Medicine, Mahidol University, 420/6 Ratchawithi Rd, Ratchathewi, Bangkok, 10400 Thailand

**Keywords:** DHA-PPQ, *Plasmodium falciparum*, *kelch13*, *Pfcrt*, *plasmepsin-2*

## Abstract

**Background:**

Dihydroartemisinin-piperaquine (DHA-PPQ) combination therapy is the current first-line treatment for *Plasmodium falciparum* malaria in Thailand. Since its introduction in 2015, resistance to this drug combination has emerged in the eastern part of the Greater Mekong Subregion including the eastern part of Thailand near Cambodia. This study aimed to assess whether the resistance genotypes have arisen the western part of country.

**Methods:**

Fifty-seven *P. falciparum*-infected blood samples were collected in Tak province of northwestern Thailand between 2013 and 2019. Resistance to DHA was examined through the single nucleotide polymorphisms (SNPs) of *kelch13*. PPQ resistance was examined through the copy number *plasmepsin-2* and the SNPs of *Pfcrt.*

**Results:**

Among the samples whose *kelch13* were successfully sequenced, approximately half (31/55; 56%) had mutation associated with artemisinin resistance, including G533S (23/55; 42%), C580Y (6/55; 11%), and G538V (2/55; 4%). During the study period, G533S mutation appeared and increased from 20% (4/20) in 2014 to 100% (9/9) in 2019. No *plasmepsin-2* gene amplification was observed, but one sample (1/54) had the *Pfcrt* F145I mutation previously implicated in PPQ resistance.

**Conclusions:**

*Kelch13* mutation was common in Tak Province in 2013–2019. A new mutation G533S emerged in 2014 and rose to dominance in 2019. PPQ resistance marker *Pfcrt* F145I was also detected in 2019. Continued surveillance of treatment efficacy and drug resistance markers is warranted.

**Supplementary Information:**

The online version contains supplementary material available at 10.1186/s12936-022-04382-5.

## Background

Malaria is a vector-borne parasitic disease threatening the health of several billions of people in the endemic areas of tropical and subtropical regions. Based on the data of 2020, 241 million people suffered from malaria in 85 malaria-endemic countries and approximately 627,000 people died from the disease worldwide [1]. The burden of malaria increased in 2020 due to the service disruption during the COVID19 pandemic. Of the 241 million cases, approximately 2% were from the Southeast Asian region, whose 60% of clinical malaria cases were due to *Plasmodium falciparum* [1].

Artemisinin-based combination therapy (ACT) was the first-line treatment for uncomplicated falciparum malaria in most malaria-endemic areas. Clinical artemisinin resistance was first identified in western Cambodia in 2008 and has spread to other countries in the Greater Mekong Subregion (GMS) [2–4]. Single nucleotide polymorphisms (SNPs) in the propeller domain of the *P. falciparum kelch13* gene were found to confer artemisinin resistance [5]. These SNPs are widely used to monitor the emergence and spread of artemisinin resistance [5]. According to a recent systematic review, a total of 165 non-synonymous SNPs on *kelch13* have been reported globally [6]. Of these non-synonymous SNPs, 84 were from South-East Asia [6]. Among the mutations that have been associated with artemisinin resistance, C580Y, R539T, Y493H, and I543T mutations were common in Cambodia, Vietnam, and Laos, whereas the F446I, N458Y, P574L, and R561H mutations dominated in the western part of Thailand, Myanmar, and China [7].

In 2015, the dihydroartemisinin (DHA)-piperaquine (PPQ) combination replaced artemisinin-mefloquine as the first-line treatment for uncomplicated *P. falciparum* malaria in Thailand [8]. As a result of a rapid rise of artemisinin resistance in Cambodia, hence a strong pressure on its partner drug, there was substantial growth in the failure rate of DHA-PPQ treatment reaching up to 60% in some areas of Cambodia [9–11]. Amplification of *P. falciparum plasmepsin-2* and *3* genes on chromosome 14 was associated with PPQ resistance in Cambodian isolates [9, 12]. This marker has since been adopted as a molecular marker of PPQ resistance [13–15]. Recent studies also reported that mutations in *P. falciparum* chloroquine resistance transporter (*Pfcrt)* gene at nucleotide positions downstream of the 4-aminoquinoline resistance locus (amino acids 72–76), particularly at codons T93S, H97Y, F145I may also contribute to PPQ resistance [13, 16, 17].

Reports of DHA-PPQ treatment resistance have come mainly from the eastern GMS, but the therapeutic efficacy of DHA-PPQ was still high in Myanmar [18]. Thus, it is crucial to monitor DHA-PPQ resistance in the western part of Thailand, as it is the gateway to Myanmar, Bangladesh and India. It was hypothesized that the frequency of DHA-PPQ resistance may have risen in this area after the introduction of DHA-PPQ in 2015. To test this hypothesis, the molecular markers of *P. falciparum* DHA-PPQ resistance were determined in Tak province of northwestern Thailand, using specimens collected during 2013–2019. The resistance markers included a selection of well-supported SNPs on *kelch13* for artemisinin resistance and *plasmepsin-2* amplification and SNPs on *Pfcrt* for PPQ resistance.

## Methods

**Fig. 1:**
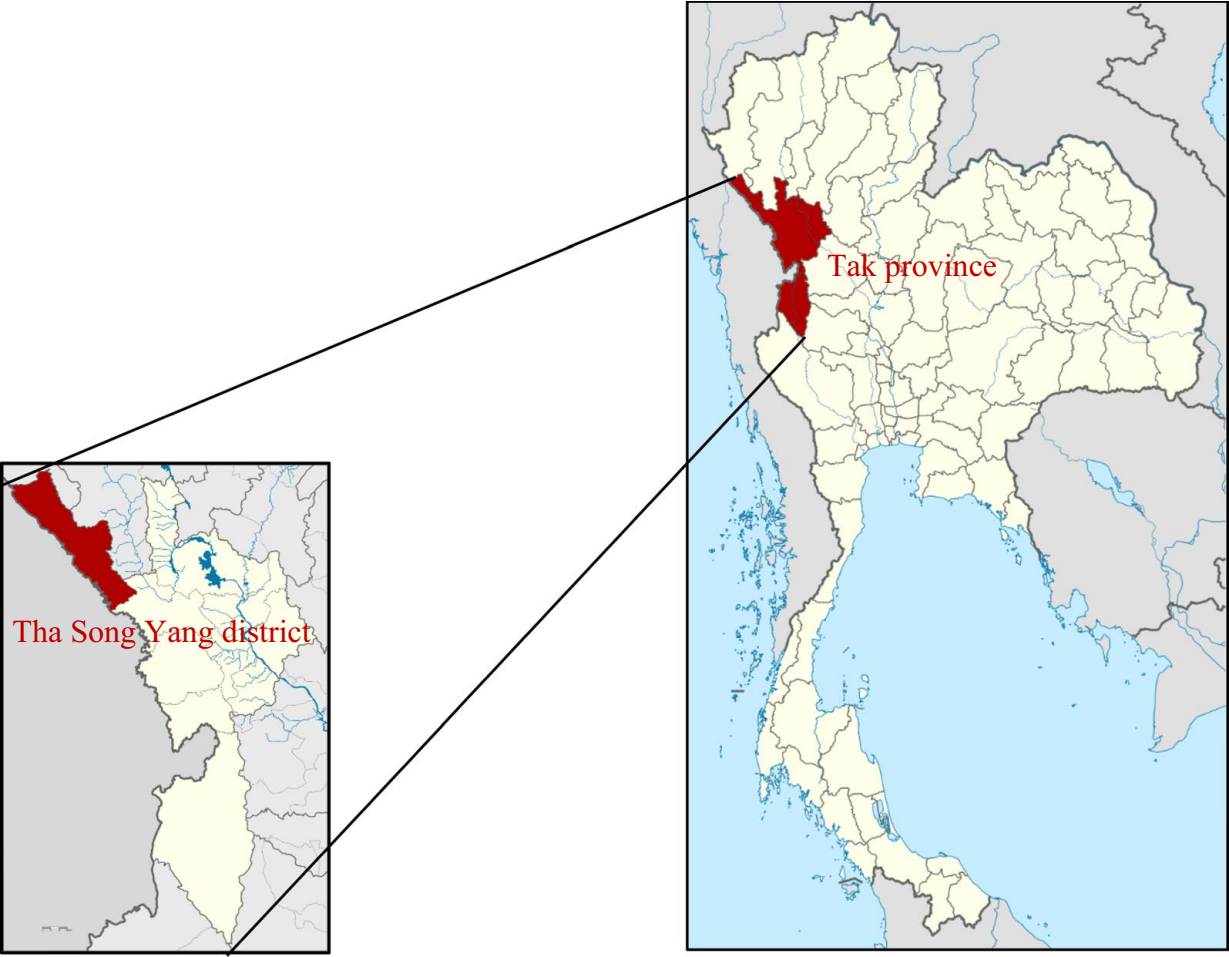
Map showing Tha Song Yang district, Tak province, Thailand [32, 33]

This is a retrospective analysis of molecular markers of artemisinin and PPQ resistance in 57 *P. falciparum* specimens. The parasites were collected as dry blood spots (DBS) or frozen whole blood between 2013 and 2019 from Tha Song Yang district of Tak Province (Fig. 1) in northwestern Thailand.

### DNA extraction

Genomic DNA of *P. falciparum*-infected blood was extracted from DBS or frozen whole blood samples using QIAamp DNA Mini Kit or Qiagen DNeasy Blood & Tissue Kit following the manufacturer’s instructions.

### Determining *kelch13* mutations

Nested polymerase chain reaction (PCR) was carried out according to the K13 Artemisinin Resistance Multicenter Assessment (KARMA) standard operating procedure [7]. The primary and secondary forward and reverse primers can be found in Additional file 1: Table S1 [7]. Sequencing was carried out using the commercial dye-terminator method and the sequences were aligned against the 3D7 reference strain to detect mutations in the propeller domain between codons 442 and 687.

### Determining *plasmepsin-2* copy number

The copy number of the *plasmepsin-2* gene per genome was determined by SYBR Green quantitative PCR (qPCR) (Additional file 1: Table S2). *Plasmodium falciparum ꞵ-tubulin*, a single copy gene, was used as the reference for copy number quantification. The primer sequences for both *plasmepsin-2* and *ꞵ-tubulin* qPCR (Additional file 1: Table S1) were from Witkowski et al. [9]. The copy number of *plasmepsin-2* was estimated as 2^−dCt^ where dC_t_ was the deviation of C_t,*plasmepsin-2*_ from the *plasmepsin-2*/*β*-*tubulin* trendline constructed from serial dilution of cultured 3D7, which has a single copy of *plasmepsin-2* per genome. A copy number value of 1.5 or higher was considered multicopy. All qPCRs were performed in duplicates. *P. falciparum* parasite isolates obtained from northeastern Thailand, where multicopy *plasmepsin-2* was common, were used to provide a reference for comparison.

### Determining *Pfcrt* mutations

Primers (Additional file 1: Table S1) were used to amplify gene fragments containing the T93S and H97Y locus in exon 2, and the F145I locus in exon 3. *Pfcrt* exon 2 and exon 3 amplification was carried out according to the PCR conditions in Additional file 1: Table S3. Sequencing was performed using the commercial dye-terminator method and the results were aligned against the 3D7 reference sequence to detect mutations.

### Data analysis

The histogram was prepared using Microsoft Excel. Fisher’s exact test (SPSS version 18) was used to compare the proportions of G533S between 2014–2015 and 2018–2019.

## Results

### G533S mutation of *kelch13* emerged and became the dominant genotype in Tak Province

Fifty-seven *P. falciparum* specimens were collected from Tha Song Yang district, Tak province (Fig. 1) during 2013–2019. Of these, 55 were successfully amplified and sequenced. Mutations were observed in 31 samples (56%). The most common mutation was G533S (23/55; 42%) which has been shown to reduce in vitro artemisinin sensitivity in a previous study [19]. G533S was first detected in 2014 (Table 1), but by 2019, all isolates had this mutation. Comparing years 2014–2015 to 2018–2019, the proportions of parasites with G533S increased from 30% (11/37) to 80% (12/15) (p = 0.002, Fisher’s exact test). Two additional resistance-associated mutations C580Y (6/55, 11%) and G538V (2/55; 4%) were found, but no other major mutations (P441L, F446I, G449A, N458Y, C469F, M476I, A481V, Y493H, P527H, N537I, R539T, I543T, P553L, R561H, V568G, P574L, F673I, A675V) were detected. One isolate had the R659R synonymous SNP and one had both G533S and A564A.

**Table 1:** Non-synonymous SNPs in *kelch13* found in Tha Song Yang district, Tak province, Thailand

SNPs	2013 (n = 2)	2014 (n = 20)	2015 (n = 17)	2016 (n = 1)	2017 (n = 0)	2018 (n = 6)	2019 (n = 9)
G533S^a^	0	4 (20%)	7 (41%)	0	0	3 (50%)	9 (100%)
C580Y^b^	2 (100%)	2 (10%)	0	0	0	2 (33.3%)	0
G538V^c^	0	1 (5%)	1 (5.9%)	0	0	0	0

^a^Associated with in vitro artemisinin resistance in Zhang’s study [19]

^b^Validated as artemisinin resistance

^c^Associated with artemisinin resistance

### No evident marker of PPQ resistance was found

**Fig. 2:**
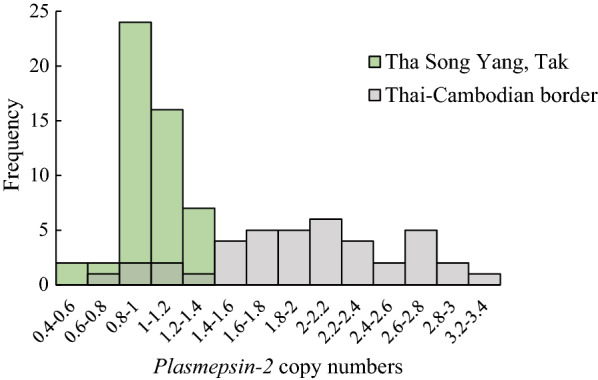
The *plasmepsin-2* copy number distribution of *P. falciparum* isolates from Tha Song Yang district, Tak province (green). The distribution is overlaid by that of isolates from Srisaket Province on the Thai-Cambodian border (grey). The Srisaket samples were collected from malaria patients during 2015 to 2017

Two markers of PPQ resistance were examined, *plasmepsin-2* amplification and *Pfcrt* mutations (T93S, H97Y, and F145I). Fifty-one isolates were successfully analysed for the *plasmepsin-2* copy number, but none had multicopy *plasmepsin-2* (Fig. 2). Fifty-two isolates were successfully sequenced at the *Pfcrt* T93S and H97Y locus, and neither mutation was found. Of the 54 isolates successfully sequenced at the F145I locus, one had this mutation. This parasite was collected in 2019 and also had the G533S mutation. The complete genotyping results can be found in Table 2.

**Table 2:** Summary of DHA-PPQ resistance markers in test samples

Isolate ID	Year of collection	*Kelch13* SNPs	*Plasmepsin-2* CNV		*Pfcrt* SNPs			
			Copy number	Classification	Exon 2		Exon 3	
					T93S	H97Y	F145I	Other
1	2013	C580Y	1.2	Single	T93	H97	F145	No SNPs
2	2013	C580Y	0.7	Single	T93	H97	F145	No SNPs
3	2014	–	1.1	Single	T93	H97	F145	No SNPs
4	2014	No SNPs	1.1	Single	T93	H97	F145	No SNPs
5	2014	No SNPs	1.1	Single	T93	H97	F145	No SNPs
6	2014	No SNPs	1.1	Single	T93	H97	F145	No SNPs
7	2014	R659R	1.0	Single	T93	H97	F145	No SNPs
8	2014	C580Y	1.3	Single	T93	H97	F145	No SNPs
9	2014	C580Y	0.9	Single	T93	H97	F145	No SNPs
10	2014	No SNPs	0.6	Single	T93	H97	F145	No SNPs
11	2014	No SNPs	1.2	Single	T93	H97	F145	No SNPs
12	2014	No SNPs	1.2	Single	T93	H97	F145	No SNPs
13	2014	No SNPs	1.2	Single	-	-	F145	No SNPs
14	2014	No SNPs	0.9	Single	T93	H97	F145	No SNPs
15	2014	G538V	1.1	Single	T93	H97	F145	No SNPs
16	2014	G533S	1.1	Single	T93	H97	F145	No SNPs
17	2014	G533S	0.9	Single	T93	H97	F145	No SNPs
18	2014	No SNPs	1.1	Single	T93	H97	F145	No SNPs
19	2014	No SNPs	1.3	Single	T93	H97	–	–
20	2014	G533S	1.1	Single	T93	H97	F145	No SNPs
21	2014	G533S	1.3	Single	T93	H97	F145	No SNPs
22	2014	No SNPs	0.9	Single	T93	H97	F145	No SNPs
23	2014	No SNPs	1.0	Single	T93	H97	F145	No SNPs
24	2015	G533S	1.0	Single	–	–	F145	No SNPs
25	2015	G533S	1.0	Single	T93	H97	F145	No SNPs
26	2015	No SNPs	0.9	Single	T93	H97	F145	No SNPs
27	2015	No SNPs	0.9	Single	T93	H97	F145	No SNPs
28	2015	G533S	0.8	Single	T93	H97	F145	No SNPs
29	2015	No SNPs	1.0	Single	T93	H97	F145	No SNPs
30	2015	–	1.4	Single	T93	H97	F145	No SNPs
31	2015	G533S	0.8	Single	T93	H97	F145	No SNPs
32	2015	G538V	0.9	Single	T93	H97	F145	No SNPs
33	2015	No SNPs	–	–	T93	H97	–	–
34	2015	G533S	1.0	Single	T93	H97	F145	No SNPs
35	2015	G533S	0.9	Single	T93	H97	F145	No SNPs
36	2015	No SNPs	0.9	Single	T93	H97	F145	No SNPs
37	2015	No SNPs	0.9	Single	T93	H97	F145	No SNPs
38	2015	No SNPs	0.5	Single	T93	H97	F145	No SNPs
39	2015	No SNPs	0.9	Single	-	-	F145	No SNPs
40	2015	No SNPs	1.2	Single	T93	H97	F145	No SNPs
41	2015	G533S	1.1	Single	T93	H97	F145	No SNPs
42	2016	No SNPs	1.2	Single	T93	H97	F145	No SNPs
43	2018	G533S	0.9	Single	T93	H97	F145	No SNPs
44	2018	No SNPs	0.8	Single	T93	H97	F145	No SNPs
45	2018	C580Y	1.0	Single	T93	H97	F145	No SNPs
46	2018	C580Y	1.1	Single	T93	H97	F145	No SNPs
47	2018	G533S	–	–	–	–	–	–
48	2018	G533S	0.8	Single	T93	H97	F145	No SNPs
49	2019	G533S A564A	–	–	–	–	145I	S140P
50	2019	G533S	–	–	T93	H97	F145	No SNPs
51	2019	G533S	1.0	Single	T93	H97	F145	No SNPs
52	2019	G533S	–	–	T93	H97	F145	No SNPs
53	2019	G533S	0.9	Single	T93	H97	F145	No SNPs
54	2019	G533S	–	–	T93	H97	F145	No SNPs
55	2019	G533S	0.8	Single	T93	H97	F145	No SNPs
56	2019	G533S	0.8	Single	T93	H97	F145	No SNPs
57	2019	G533S	1.1	Single	T93	H97	F145	No SNPs

No data

## Discussion

Over the last decade, there has been remarkable progress in controlling *P. falciparum* malaria in the Greater Mekong subregion [1]. Likewise, in Thailand the reported case number of *P. falciparum* malaria declined from 15,740 in 2013 to 638 in 2019 [20]. In Tha Song Yang district, the annual *P. falciparum* malaria case numbers were 2388, 941, 352, 68, 17, 53, and 27 during 2013–2019 [20]. However, this achievement is still under the risk of resistance to antimalarials. In 2008–2009, slow parasite clearance after artemisinin therapy of falciparum malaria was first documented in western Cambodia, and presumed to reflect emerging artemisinin resistance [2, 3]. Since then, artemisinin resistance has spread or emerged independently throughout the Greater Mekong subregion [4, 10, 21]. As a result of the falling efficacy of artemisinin-mefloquine in Thailand, DHA-PPQ was introduced as the first-line treatment in 2015 [8, 22]. However, treatment failure soon appeared in the eastern border region of Thailand, coinciding with observations of PPQ resistance in Cambodia [11, 13, 23–25]. At present, markers of resistance to artemisinin and PPQ have been identified [5, 9, 12, 16, 17, 26].

*Kelch13* mutations are the most well-established markers of artemisinin resistance [21, 27]. It has been shown that whereas C580Y was the main mutation in the eastern GMS, F446I was more common in Myanmar [10]. C580Y has now been found in many parts of Thailand but has become near fixation along the Cambodian border [4, 10, 15, 28]. The distributions of other *kelch13* alleles in the country vary geographically—with Y493H and R539T found mainly in the east, P574L in the west, and R561H in northwest [28]. In this study, a new mutation, G533S, was detected in 2014 and became the most common resistance mutation in the study site. This mutation was not detected in a previous study from the area in 2012–2014 [28]. Despite this, the timing of its first detection in 2014 and the study site’s proximity to Myanmar and China agreed with the G533S appearance on the China-Myanmar border at around the same time [19]. Importantly, the frequency of G533S rose significantly, and in 2019 all 9 specimens examined had this mutation. Although this mutation has not been formally associated with clinical resistance, parasites carrying this mutation were recently shown to have elevated ring-stage survival compared to the wild-type parasite [19].

Amplification of *plasmepsin-2* is widely used as a marker to trace PPQ resistance [9, 12–15]. Previous studies suggested that this genotype was restricted to eastern GMS [10, 14, 15]. Consistent with these reports, none of the *P. falciparum* isolates in Tak province from 2013 to 2019 had *plasmepsin-2* amplification.

The second molecular marker of PPQ resistance is *Pfcrt* [16, 29]. Several mutations including T93S, H97Y, and F145I were implicated in treatment failure [13, 26]. The frequencies of these mutations have risen in eastern GMS [14, 15], but remained much less prevalent in the Thai-Myanmar border region [30]. In the present study, no parasite had T93S and H97Y and only one parasite had F145I. Because the F145I mutation has been shown to confer a high level of resistance of PPQ through transfection [16] and be associated with a five-fold increased risk of DHA-PPQ treatment failure [26], the detection of this mutation raises concern over its potential future establishment in the area.

In summary, this study detected for the first time the *kelch13* G533S mutation in Tak province of western Thailand. This mutation has been associated with artemisinin resistance in vitro [19] and its increase in frequencies was observed during 2014–2019. In addition, although no *plasmepsin-2* amplification was observed, the PPQ resistance *Pfcrt* F145I mutation was detected in a parasite that also carried the G533S mutation. Given the threat of drug resistance, close monitoring of resistance markers and treatment failure in the area is well warranted.

## Limitations

The study involved a small number of samples—55 for *kelch13* mutation, 51 for *plasmepsin-2* amplification, and 54 for *Pfcrt* mutation—from one district of Tak Province, Thailand. The low number of samples per year, particularly from 2016 onwards, was due to the low incidence of *P. falciparum* malaria in the study site. As such, the study has limited power to detect rare mutations and the results may not be representative of the broader area of western Thailand. In addition, although the *kelch13* G533S was first detected in 2014, its first emergence in the study area may have been earlier.

## Conclusions

Thailand is eliminating malaria, but the development of resistance against the current national first-line drug DHA-PPQ would hinder the progress. Indeed, in Srisaket and Ubon Ratchathani provinces in Northeastern Thailand, the first-line treatment had to be changed to pyronaridine-artesunate (Pyramax™) in 2019 due to the decreased efficacy of DHA-PPQ [31]. For the northwestern area in this study, the predominance of the *kelch13* G533S could be a warning sign of developing resistance. Thus, the resistance markers and treatment efficacy in this area should be closely monitored.

## Supplementary Information

**Additional file 1: Table S1.** Primers used in analysis of DHA-PPQ resistance markers. **Table S2.** Thermocycling condition of *plasmepsin-2* amplification by SYBR Green real-time PCR. **Table S3.** Thermocycling condition of exon 2 and exon 3 of *Pfcrt* gene amplification.

## Data Availability

All data generated or analysed during this study are included in this published article and its additional files.

## Abbreviations

DBS: Dry blood spots
CNV: Copy number variation
DHA: Dihydroartemisinin
*Pfcrt*: *P. falciparum* chloroquine resistance transporter
PPQ: Piperaquine
SNPs: Single nucleotide polymorphisms
