# Supplementary material for: Molecular markers of dihydroartemisinin-piperaquine resistance in northwestern Thailand
Source: Malar J. 2022 Nov 27;21:352. doi: 10.1186/s12936-022-04382-5 (PMC9701414; doi:10.1186/s12936-022-04382-5)
Supplement: Supplementary file 1 — Additional file 1: Table S1. Primers used in analysis of DHA-PPQ resistance markers. Table S2. Thermocycling condition of plasmepsin-2 amplification by SYBR Green real-time PCR. Table S3. Thermocycling condition of exon 2 and exon 3 of Pfcrt gene amplification. [file 12936_2022_4382_MOESM1_ESM.docx]

**Additional file 1**

**Determining *kelch13* mutations**

*Kelch13* sequencing was performed according to the K13 Artemisinin Resistance Multicenter Assessment (KARMA) standard operation procedure with some refinement [7]. Primers (Table S1) from the KARMA project were used to amplify the gene fragment for sequencing [7]. The primary PCR was carried out in 25 μl and contained 2X Gotaq green PCR mix, 0.25 μM of each “K13_PCR_F” forward and “K13_PCR_R” reverse primer, the DNA template, and nuclease free distilled water. The secondary PCR was carried out in 50 μl, containing 2X Gotaq green PCR mix, 0.25 μM of each “K13_N1_F” forward and “K13_N1_R” reverse primer, 5 μl of the primary PCR product and nuclease free distilled water.

**Table S1.** Primers used in analysis of DHA-PPQ resistance markers

| **Primer name** | **Amplification** | **5'-Sequence-3'** |
| --- | --- | --- |
| K13_PCR_F | *kelch13* primary PCR | CGGAGTGACCAAATCTGGGA |
| K13_PCR_R |  | GGGAATCTGGTGGTAACAGC |
| K13_N1_F | *kelch13* nested PCR | GCCAAGCTGCCATTCATTTG |
| K13_N1_R |  | GCCTTGTTGAAAGAAGCAGA |
| *PfPM2*_CN_F | *plasmepsin-2* CNV | TGGTGATGCAGAAGTTGGAG |
| *PfPM2*_CN _R |  | TGGGACCCATAAATTAGCAGA |
| *Pfβ-tubulin*_CN_F | *plasmepsin-2* CNV | TGATGTGCGCAAGTGATCC |
| *Pf β-tubulin*_CN_R |  | TCCTTTGTGGACATTCTTCCTC |
| *Pfcrt*_exon2_F | *Pfcrt* exon 2 | TTAAGTATTATTTATTTAAGTGTATGTG |
| *Pfcrt*_exon2_R |  | GATTTATCTTACTTTTGAATTTCCC |
| *Pfcrt*_exon3_F | *Pfcrt* exon 3 | GACACCGAAGCTTTAATTTAC |
| *Pfcrt*_exon3_R |  | GAACATATTAATAGGAATACTTAATTG |

**Assessment of *plasmepsin-2* copy numbers**

The *plasmepsin-2* copy number per genome was measured by SYBR quantitative PCR (qPCR). *Plasmepsin-2* (PF3D7_1408000) was amplified from the position 423 to 501. The single copy *β-tubulin* was used as the reference. *β*-*tubulin* (PF3D7_1008700) was amplified from the position 1,246 to 1,324. The qPCR was carried out in 20 μl containing AccuPower 2X GreenStar^TM^ qPCR master mix or RealMOD^TM^ Green W^2^ 2X qPCR mix, 0.25 μM of each forward and reverse primer, 4 μl of the DNA template and distilled water. Amplification was performed according to the conditions in Table S2.

**Table S2.** Thermocycling condition of *plasmepsin-2* amplification by SYBR Green real-time PCR

| Cycle | Temperature | Time | No. of cycles |
| --- | --- | --- | --- |
| Initial denaturation | 95℃ | 5 min | 1 |
| Denaturation | 95℃ | 15 sec | 45 |
| Annealing and extension | 60℃ | 20 sec |  |
| Melting | 65-95℃ increased by 0.5℃ per 0.05 sec | | |

**Determining *Pfcrt* mutations on exons 2/3**

*Pfcrt* exon 2 and exon 3 amplification was carried out in 25 μl using 2X GoTaq® Green Master Mix, 0.32 μM of each forward and reverse primer, the DNA template, and nuclease free water. Because of the limited quantity of the parasite DNA and the need for multiple sequencing runs, the PCR products for both exon 2 and exon 3 were amplified in two rounds. The second round of PCR used the same primers, 0.1-3 μl of the primary PCR product as the template, and 15 or 20 amplification cycles.

**Table S3.** Thermocycling condition of exon 2 and exon 3 of *Pfcrt* gene amplification

| PCR | Cycle | Exon 2 | | | Exon 3 | | |
| --- | --- | --- | --- | --- | --- | --- | --- |
|  |  | Temperature | Time | No. of cycles | Temperature | Time | No. of cycles |
| Primary PCR | Initial denaturation | 95℃ | 5 min | 1 | 95℃ | 5 min | 1 |
|  | Denaturation | 95℃ | 30 sec | 40 | 95℃ | 30 sec | 40 |
|  | Annealing | 53℃ | 45 sec |  | 53℃ | 45 sec |  |
|  | Extension | 60℃ | 45 sec |  | 60℃ | 45 sec |  |
|  | Final extension | 60℃ | 10 min | 1 | 60℃ | 10 min | 1 |
| Second PCR | Initial denaturation | 95℃ | 5 min | 1 | 95℃ | 5 min | 1 |
|  | Denaturation | 95℃ | 30 sec | 15 | 95℃ | 30 sec | 20 |
|  | Annealing | 53℃ | 45 sec |  | 53℃ | 45 sec |  |
|  | Extension | 60℃ | 45 sec |  | 60℃ | 45 sec |  |
|  | Final extension | 60℃ | 10 min | 1 | 60℃ | 10 min | 1 |
